# Glycemic changes after vitamin D supplementation in patients with type 1 diabetes mellitus and vitamin D deficiency

**DOI:** 10.4103/0256-4947.72265

**Published:** 2010

**Authors:** Khalid S. Aljabri, Somoa A. Bokhari, Murtadha J. Khan

**Affiliations:** From the Division of Endocrinology, Department of Medicine, King Fahad Armed Forces Hospital, Jeddah, Saudi Arabia

## Abstract

**BACKGROUND:**

A prospective, nonblinded and nonrandomized controlled trial was conducted to test the hypothesis that vitamin D supplementation would improve glycemic control in patients with type 1 diabetes mellitus who have vitamin D deficiency.

**PATIENTS AND METHODS:**

Eighty patients with type 1 diabetes mellitus who had 25-hydroxyvitamin D levels less than 50 nmol/L were assigned to receive 4000 IU of vitamin D3. Calcium supplements were provided to ensure a total calcium intake of 1200 mg/d. Glycosylated hemoglobin and 25-hydroxyvitamin D levels were measured at baseline and at 12 weeks.

**RESULTS:**

There was a significant difference in mean (SD) glycosylated hemoglobin level (%) between the groups that achieved 25-hydroxyvitamin D levels of <35.4 nmol/L, 35.4-51 nmol/L and >51 nmol/L at 12 weeks (*P*=.02). There was a significant difference in glycosylated hemoglobin change from baseline between the groups that achieved 25-hydroxyvitamin D levels of <35.4 nmol/L, 35.4-51 nmol/L and >51 nmol/L at 12 weeks (*P*=.04). There was a significant difference in 25-hydroxyvitamin D level between the groups that achieved glycosylated hemoglobin levels of <7.8, 7.8-9.9 and >9.9 at 12 weeks (*P*=.001). Patients were more likely to achieve lower glycosylated hemoglobin levels at 12 weeks if they had higher 25-hydroxyvitamin D levels at 12 weeks (r=-0.4, *P*=.001).

**CONCLUSIONS:**

There was an observed effect of vitamin D supplementation on glycemic control in vitamin D-replete, type 1 diabetes mellitus patients. Further studies are needed to determine if these findings are applicable.

Type 1 diabetes mellitus results from cellular-mediated autoimmune destruction of the beta cells of the pancreas.^1^ Serum 25-hydroxyvitamin D (25-OHD) concentrations are largely determined by environmental factors, mainly through vitamin D intake and ultraviolet exposure. The sun is the primary source of vitamin D, which is synthesized endogenously in skin to produce cholecalciferol (vitamin D3), although a small proportion (<20%) of vitamin D comes through diet from a limited range of foods (in the form of ergocalciferol [vitamin D2] and vitamin D3).^2^ The main marker of vitamin D status is the metabolite 25-OHD, which is synthesized in the liver.^3^,^4^

A relationship between type 1 diabetes mellitus and vitamin D deficiency has been reported.^5^,^6^ The prevalence of vitamin D deficiency in patients with type 1 diabetes was 15% to 90.6%.^7^–^9^ There is evidence that vitamin D is important in the prevention of islet cell death and might be useful in improving the survival of islet cell grafts, and it improves the production of insulin. Low vitamin D levels were shown to have a negative effect on beta-cell function.^3^,^4^ Regular doses of vitamin D early in life have been shown to reduce the risk of developing type 1 diabetes.^5^ Vitamin D treatment has also been shown to improve glycemic control and insulin sensitivity in people with type 1 and type 2 diabetes and in normal individuals. Increasing vitamin D levels from 25 to 75 nmol/L results in a 60% improvement in insulin sensitivity.^3^,^4^,^10^–^12^ These effects have been mainly attributed to the immunomodulatory actions of vitamin D.^5^

We conducted a prospective, nonblinded and nonrandomized controlled trial to test the hypothesis that vitamin D supplementation would improve glycemic control in patients with type 1 diabetes who are vitamin D deficient.

## PATIENTS AND METHODS

Patients aged more than 12 years who had type 1 diabetes mellitus were eligible.^1^ Exclusion criteria were a history of liver disease, abnormal renal function, current use of vitamin D or calcium and pregnancy or breastfeeding. The doses of insulin were adjusted during the study. All patients were recruited from the King Fahad Armed Forces Hospital Diabetes Centre between January 2008 and June 2009. All patients gave their or their guardians’ informed consent prior to inclusion. The study was approved by the ethical board of King Fahad Armed Forces Hospital. Eligible patients met the investigators 4 weeks before the start of the study for a complete history and physical examination and to have baseline laboratory assessments, including glycosylated hemoglobin, 25-OHD, calcium, phosphorus, magnesium and parathyroid hormone. The serum level of 25-OHD was measured by competitive protein-binding assay using appropriate kits (Immunodiagnostic, Bensheim, Germany). Glycosylated hemoglobin was measured using high-performance liquid chromatography.

Vitamin D deficiency was defined as a 25-OHD level of less than 50 nmol/L.^13^ At entry into the study, patients having 25-OHD <50 nmol/L were assigned to receive 4000 IU of vitamin D and a total calcium intake of 1200 mg/d. All patients continued their current doses of insulin. The endpoints for glycemic control and vitamin D status were comparison of the glycosylated hemoglobin and 25-OHD levels at the beginning and at 12 weeks. Serum 25-OHD tertiles were defined as follow: first tertile (<35.4 nmol/L), second tertile (35.4-51 nmol/L) and the third tertile (>51 nmol/L). Glycosylated hemoglobin tertiles were defined as follows: first tertile (<7.8), second tertile (7.8-9.9%) and the third tertile (>9.9%).

Univariate analyses of baseline and follow-up demography and clinical laboratory endpoints were accomplished using the unpaired *t* test and paired *t* test where appropriate. The chi-square test was used for categorical data comparison. Pearson correlation coefficients between continuous variables were used as a measure of association. The data were analyzed by one-way analysis of variance (ANOVA), followed by post hoc LSD multiple comparison test to estimate the significance of difference between groups, and linear regression analysis was used as appropriate. All statistical analyses were performed using SPSS version 16.0. All P values were based on two-sided tests. The difference between groups was considered significant when *P* was <.05.

## RESULTS

Eighty patients completed the study. Baseline characteristics (**Table 1**) were similar for each serum 25-OHD tertile (**Table 2**). There was also a significant difference in mean glycosylated hemoglobin level between the groups that achieved 25-hydroxyvitamin D levels of <35.4 nmol/L, 35.4-51 nmol/L and >51 nmol/L at 12 weeks (*P*=.02). There was a significant difference in glycosylated hemoglobin change from baseline for each tertile. The correlation of baseline and follow-up glycosylated hemoglobin levels in patients who achieved 25-hydroxyvitamin D levels of >51 nmol/L was -1.0 (95% C.I., -1.6, -0.5), *P*=.001, Pearson test. Mean (SD) glycosylated hemoglobin levels in patients who achieved 25-hydroxyvitamin D levels of >75 nmol/L as compared to those who achieved <75 nmol/L at 12 weeks were 7.3(1.5) vs. 9.1(2.4, respectively, *P*=.02. Patients were more likely to achieve lower glycosylated hemoglobin if they had higher 25-hydroxyvitamin D levels at 12 weeks (r= -0.4, *P*=.001, Pearson’s test).

**Table 1:** Baseline characteristics of study population.

Numbers	80
Male / Female	29 (36.3) / 51 (63.7)
Age (years)	21.0 (7.6)
Body mass index (kg /m^2^)	22.3 (4.0)
Diabetes duration (years)	7.1 (6.7)
Calcium (mmol/L)	2.3 (0.2)
Phosphorus (mmol/L)	1.3 (0.3)
Magnesium (mmol/L)	0.8 (0.1)
Parathyroid hormone (ng/L)	5.8 (6.1)
Glycosylated hemoglobin at base line (%)	9.4 (2.3)
25-hydroxyvitamin D level (nmol/L)	
<25	56 (70)
≥25	24 (30)

Data are mean (standard deviation) or number (percent).

**Table 2:** Baseline characteristics by 25-hydroxy vitamin D tertiles.

Parameters	25-hydroxyvitamin D level (nmol/L)			*P* value
Values	<35.4	35.4-51	>51	
Numbers (%)	26 (32.5)	27 (33.8)	27 (33.8)	
Male/Female (%)	27.6/35.3	29.7/41.2	51.7/23.5	.03
Age (years)	19.4 (5.1)	20.5 (8.7)	23.0 (8.3)	.2
Body mass index (kg /m^2^)	21.9 (4.7)	22.5 (4.0)	22.6 (3.4)	.6
Diabetes Duration (years)	8.4 (7.3)	7.1 (6.7)	6.5 (6.5)	.7
Calcium	2.4 (0.2)	2.3 (0.1)	2.3 (0.3)	.7
Phosphorus	1.3 (0.4)	1.3 (0.3)	1.3 (0.3)	1.0
Magnesium	0.8 (0.1)	0.8 (0.1)	0.8 (0.1)	.5
Parathyroid hormone	5.7 (4.4)	5.4 (2.7)	6.1 (9.2)	.9
Glycosylated hemoglobin				
Baseline	9.8 (2.2)	9.2 (2.4)	9.2 (2.4)	.6
Follow-up	10.0 (2.1)	9.0 (2.3)	8.2 (2.3)	.02
Change (95% CI)	-0.2 (-0.5, 0.8)	-0.2 (-1.1, 0.6)	-1.0 (-1.6, -0.5)	.04
25-hydroxyvitamin D level at follow-up (nmol/L)	24.5 (5.5)	44.6 (4.4)	75.1 (17.7)	<.0001

Data are mean (standard deviation) or number (percent).

There was a significant difference in achieved 25-hydroxyvitamin D level between the groups that achieved glycosylated hemoglobin levels of <7.8, 7.8-9.9 and >9.9 at 12 weeks—61.9(23.4), 45.1(22.1) and 38.5(19.3), respectively, *P*=.001. Age and 25-hydroxyvitamin D levels at 12 weeks were significantly correlated in a linear regression with better glycosylated hemoglobin levels (–0.3, *P*=.006; and –0.4, *P*=.001, respectively). There were no significant differences in the mean and the tertiles of glycosylated hemoglobin between the groups that achieved 25-hydroxyvitamin D levels of <35.4 nmol/L, 35.4-51 nmol/L and >51 nmol/L at 12 weeks in both genders (**Tables 3 and 4**).

**Table 3:** Glycosylated hemoglobin tertiles by 25-hydroxyvitamin D tertiles at 12 weeks.

		Glycosylated hemoglobin tertiles (%)								
		>9.9			7.8-9.9			<7.8		
		Female	Male	Total	Female	Male	Total	Female	Male	Total
25-hydroxyvitamin D tertiles (nmol/L)	<35.4	50	50	50	33.3	37.5	34.6	16.7	12.5	15.4
	35.4-51	38.1	16.7	33.3	33.3	66.7	40.7	28.6	16.7	25.9
	>51	25	13.3	18.5	25	26.7	25.9	50	70	55.6

**Table 4:** Tertiles at 12 weeks by gender.

25-hydroxyvitamin D tertiles (nmol/L)	Glycosylated hemoglobin	Female	Male	*P* Value
<35.4	Baseline	9.8 (2.4)	9.8 (1.9)	.9
	Follow-up	10.1 (2.4)	9.7 (1.4)	.96
	Mean change	0.4	- 0.1	.5

35.4-51	Baseline	9.3 (2.6)	9.1 (1.7)	.9
	Follow-up	9.1 (2.5)	8.8 (1.6)	.8
	Mean change	0.2	- 0.3	.9

>51	Baseline	9.8 (2.2)	8.8 (2.5)	.3
	Follow-up	8.2 (2.7)	8.2 (2.1)	.9
	Mean change	-1.6	- 0.6	.05

Values are mean (standard deviation)

## DISCUSSION

Diabetes mellitus has been recognized as a main independent risk factor for cardiovascular diseases.^14^ Clinical studies indicate that most diabetic patients die due to cardiovascular diseases, with atherosclerosis accounting for about 8% to 10% of all diabetic deaths.^15^ Diabetes mellitus is a complex, progressive disease, accompanied by multiple complications. Hyperglycemia has been accepted as being essential for the development of diabetic complications. The Diabetes Control and Complication Trial (DCCT) established that prolonged exposure to hyperglycemia is considered the primary factor associated with the development of diabetic macrovascular complications in type 1 diabetic patients.^16^ The DCCT showed that improvement of glycemic control, as measured by reduction in glycosylated hemoglobin levels, significantly reduced the risk of development and/or progression of all diabetic complications and also reduced the mortality and morbidity due to cardiovascular diseases in type 1 diabetes mellitus patients.

Insulin resistance plays a larger role in the type 1 diabetes disease process than is commonly recognized. Subsets of people with mild manifestations of the type 1 autoimmune disease process could benefit from treatments aimed at improving the insulin-resistant state.^17^ There is evidence that vitamin D is important in the prevention of islet cell death.^18^ Reports have shown the association of hypovitaminosis D with insulin resistance and beta-cell dysfunction,^3^,^19^,^20^ and vitamin D is required to improve the production of insulin.^3^,^4^ There are few studies that have examined the effect of supplementation with a variety of formulations of vitamin D on type 2 diabetes mellitus parameters. Among 18 young healthy men, supplementation with 1,25-(OH)2D3 for 7 days did not change fasting glycemia or insulin sensitivity.^21^ In another small study of 14 patients with type 2 diabetes mellitus, 1-OHD3 administration daily for 3 weeks enhanced insulin secretion, but had no effect on post-load glucose tolerance.^22^ Ljunghall et al randomized 65 middle-aged men with impaired glucose tolerance or mild diabetes and sufficient vitamin D levels at baseline to 0.75 g/d of 1-OHD3 or placebo for 3 months and found no effect on fasting or stimulated glucose tolerance.^23^ In a crossover trial, 20 patients with type 2 diabetes mellitus and vitamin D deficiency were treated for 4 days with 1,25-OHD, and no change was seen in fasting or stimulated glucose, insulin or C-peptide concentrations.^24^ Pittas et al have shown that insulin sensitivity is improved by as much as 60% when levels of vitamin D are increased from 25 to 75 nmol/L.^25^ In a post hoc analysis of a 2-year trial, supplementation with vitamin D3 or 1-OHD3 had no effect on fasting glycemia in postmenopausal nondiabetic women.^26^ One study reported that glycemic control became worse in three Asian patients following vitamin D supplementation;^27^ however, these patients received vitamin D2 and not vitamin D3. Vitamin D2 has several unknown metabolites with unknown effects, and certain vitamin D receptor genotypes are big determinants of insulin secretory capacity in various ethnic groups.^28^,^29^ Luo et al showed that among 109 patients aged over 50 years with type 2 diabetes who received cholecalciferol 2000 IU daily for 3 months, glycosylated hemoglobin concentrations and insulin use did not change significantly.^30^

The patients in our study were repleted with vitamin D3, and using up to 4000 IU of vitamin D3 to reverse states of deficiency was found to be safe.^31^ There are no studies that have examined the effect of supplementation with a variety of formulations of vitamin D on glycemic control in type 1 diabetes mellitus. We are the first to have undertaken a study that has shown that the addition of vitamin D3 to insulin therapy produces a significant improvement in glycemic control. This effect on glycemic control was sustained over a period of 12 weeks; we do not know if this effect would be sustained further.

The study had some limitations. Increased hypoglycemia is a well-known complication of improved glucose control, and an important goal of therapy is to minimize this risk. Hypoglycemic episodes were not obtained by our patients. The study was neither blinded nor randomized, and the incremental doses of insulin were not analyzed.

In conclusion, diabetes is one of the fastest-growing chronic diseases worldwide. Vitamin D deficiency is common, and repletion might improve glycemic control in type 1 diabetes. Vitamin D3 is inexpensive and readily available. Well-designed clinical studies are required to ascertain if improving 25-OHD levels from deficiency to sufficiency improves glycemic control in patients with type 1 diabetes.
